# Comprehensive behavioral analysis of pituitary adenylate cyclase-activating polypeptide (PACAP) knockout mice

**DOI:** 10.3389/fnbeh.2012.00058

**Published:** 2012-10-02

**Authors:** Satoko Hattori, Keizo Takao, Koichi Tanda, Keiko Toyama, Norihito Shintani, Akemichi Baba, Hitoshi Hashimoto, Tsuyoshi Miyakawa

**Affiliations:** ^1^Division of Systems Medical Science, Institute for Comprehensive Medical Science, Fujita Health UniversityToyoake, Aichi, Japan; ^2^Japan Science and Technology Agency, Core Research for Evolutional Science and TechnologyKawaguchi, Saitama, Japan; ^3^Center for Genetic Analysis of Behavior, National Institute for Physiological SciencesOkazaki, Aichi, Japan; ^4^Genetic Engineering and Functional Genomics Group, Frontier Technology Center, Graduate School of Medicine, Kyoto UniversityKyoto, Kyoto, Japan; ^5^Department of Pediatrics, Kyoto Prefectural University of MedicineKyoto, Kyoto, Japan; ^6^Laboratory of Molecular Neuropharmacology, Graduate School of Pharmaceutical Sciences, Osaka UniversitySuita, Osaka, Japan; ^7^United Graduate School of Child Development, Osaka University, Kanazawa University and Hamamatsu University School of MedicineSuita, Osaka, Japan

**Keywords:** pituitary adenylate cyclase-activating polypeptide, knockout mouse, behavior, hyperactivity, social interaction, working memory, mental disorder

## Abstract

Pituitary adenylate cyclase-activating polypeptide (PACAP) is a neuropeptide acting as a neurotransmitter, neuromodulator, or neurotrophic factor. PACAP is widely expressed throughout the brain and exerts its functions through the PACAP-specific receptor (PAC_1_). Recent studies reveal that genetic variants of the PACAP and PAC_1_ genes are associated with mental disorders, and several behavioral abnormalities of PACAP knockout (KO) mice are reported. However, an insufficient number of backcrosses was made using PACAP KO mice on the C57BL/6J background due to their postnatal mortality. To elucidate the effects of PACAP on neuropsychiatric function, the PACAP gene was knocked out in F1 hybrid mice (C57BL/6J × 129SvEv) for appropriate control of the genetic background. The PACAP KO mice were then subjected to a behavioral test battery. PACAP deficiency had no significant effects on neurological screen. As shown previously, the mice exhibited significantly increased locomotor activity in a novel environment and abnormal anxiety-like behavior, while no obvious differences between genotypes were shown in home cage (HC) activity. In contrast to previous reports, the PACAP KO mice showed normal prepulse inhibition (PPI) and slightly decreased depression-like behavior. Previous study demonstrates that the social interaction (SI) in a resident-intruder test was decreased in PACAP KO mice. On the other hand, we showed that PACAP KO mice exhibited increased SI in Crawley's three-chamber social approach test, although PACAP KO had no significant impact on SI in a HC. PACAP KO mice also exhibited mild performance deficit in working memory in an eight-arm radial maze (RM) and the T-maze (TM), while they did not show any significant abnormalities in the left-right discrimination task in the TM. These results suggest that PACAP has an important role in the regulation of locomotor activity, social behavior, anxiety-like behavior and, potentially, working memory.

## Introduction

Pituitary adenylate cyclase-activating polypeptide (PACAP) is a member of the vasoactive intestinal peptide (VIP)/secretin/glucagon superfamily and is widely distributed throughout the central nervous system. PACAP is also found in various peripheral organs (Vaudry et al., 2000), particularly the endocrine glands, respiratory system, gastro-intestinal tract, and urogenital tract. PACAP exerts multiple activities as a neurotransmitter, neuromodulator, and neurotrophic factor via three receptors, the PACAP-specific PAC_1_ receptor, and the two PACAP/VIP-indifferent VPAC_1_ and VPAC_2_ receptors (Arimura, 1998; Vaudry et al., 2000; Hashimoto et al., 2006). PAC_1_ is the receptor that is predominantly expressed in the brain, especially in the neocortex, the limbic system, and the brain stem (Hashimoto et al., 2006).

In a genetic linkage study, fine-scale mapping of a locus for severe bipolar mood disorder on chromosome 18p11.3 suggests that the PACAP gene, which resides at 18p11.32, is located close to a bipolar disorder risk locus (McInnes et al., 2001). Recently, genetic association studies have also shown that genetic variants of the genes encoding PACAP or PAC_1_ are associated with schizophrenia (Hashimoto et al., 2007), major depressive disorder (Hashimoto et al., 2010), and post-traumatic stress disorder (PTSD) (Ressler et al., 2011). However, a few reports do not support the association of the PACAP gene with either schizophrenia or bipolar disorder (Ishiguro et al., 2001; Lohoff et al., 2008; Koga et al., 2010). Nonetheless, several reports implicate PACAP in the regulation of the hypothalamo-pituitary-adrenal axis, which is activated by psychogenic stressors (Gray et al., 2001; Hamelink et al., 2002; Hashimoto et al., 2009; Stroth et al., 2011). Chronic unpredictable stress increases PACAP mRNA expression in the dorsal part of the bed nucleus of the stria terminalis, which is a region that mediates fear- and anxiety-like behavior (Hammack et al., 2009, 2010). Moreover, exposure of PC12 cells to PACAP increases expression of endogenous disrupted-in-schizophrenia 1 (DISC1), a potential susceptibility marker for major psychiatric diseases (Hattori et al., 2007). PACAP markedly decreases the interaction between DISC1 and the DISC1-binding zinc-finger protein (DBZ), which is suggested to be one of molecular pathways that underlies the pathogenesis of schizophrenia (Hattori et al., 2007). These studies suggest that mechanisms mediated by PACAP might be involved in the pathology of these psychiatric disorders.

PACAP knockout (KO) mice display notable behavioral abnormalities, such as hyperactivity and jumping behavior in a novel environment, increased novelty-seeking behavior (Hashimoto et al., 2001), impaired prepulse inhibition (PPI) (Tanaka et al., 2006), increased immobility in the Porsolt forced swim (PS) test (Hashimoto et al., 2009), and decreased social interaction (SI) (Ishihama et al., 2010). Amphetamine and an atypical antipsychotic attenuate hyperactivity and PPI deficits in PACAP KO mice (Tanaka et al., 2006; Hashimoto et al., 2007). In addition, increased immobility in the PS test is reduced by antidepressant treatment of PACAP KO mice (Hashimoto et al., 2009). Furthermore, rearing in an enriched environment from 4 to 8 weeks of age ameliorates the hyperactivity, jumping behavior, depression-like behavior, and deficits in social behavior (Ishihama et al., 2010). It is also of note that PAC_1_ receptor-deficient mice exhibit hyperactivity, decreased anxiety-like behavior (Otto et al., 2001b), and aberrant social and sexual behaviors; for example, excessive mounting and reduced aggression (Nicot et al., 2004). These findings suggest that PACAP signaling might be involved in the pathogenesis of multiple mental disorders, such as bipolar disorder, major depressive disorder, schizophrenia, PTSD, and attention-deficit/hyperactivity disorder (ADHD).

Previous studies demonstrate that genetic variability between inbred mouse strains may affect their behavioral phenotype (Crawley et al., 1997; Matsuo et al., 2010), and that the differences observed between KO and control mice might be due to background genes linked to the targeted locus rather than to the null mutation itself (Gerlai, 1996; Silva et al., 1997; Crusio, 2004). In previous behavioral studies, F2-, F3-generation, or outbred mice were used, because the high postnatal mortality in PACAP KO mice (Gray et al., 2001; Shintani et al., 2003) makes it difficult to perform a sufficient number of backcrosses onto a C57BL/6J background, a donor strain of the mutant mice, for behavioral studies. Therefore, to assess the possible utility of PACAP KO mice as an animal model for psychiatric disorders, the background strain was tightly controlled in the present study by using F1 hybrids of the C57BL/6J and 129SvEv strains for production of KO mice. Hybrid crosses can reduce the effect of closely-linked genes flanking the targeted locus, which might be responsible for behavioral abnormalities ascribed to the null mutation (Gerlai, 1996; Silva et al., 1997; Crusio, 2004). These mice were then subjected to a comprehensive battery of behavioral tests (Takao and Miyakawa, 2006a; Takao et al., 2007). The battery of behavioral tests included neurological screening, light/dark transition (LD), open field (OF), elevated plus-maze (EP), SI, rotarod (RR), hot plate (HP), acoustic startle response/PPI, PS, RM, TM, and HC activity tests. Home cage monitoring tests and multiple paradigms for SI and memory function were used to further analyze the roles of PACAP in abnormal behavior considered to be relevant to psychiatric disorders. The behavioral tests that were used for phenotyping of PACAP KO mice in previous studies were also included in this test battery. It is important to reproduce these results, as they are susceptible to statistical false-positives or -negatives due to multiple testing and could be easily affected by genetic backgrounds (Gerlai, 1996; Crawley et al., 1997; Silva et al., 1997; Crusio, 2004; Matsuo et al., 2010), experimental conditions, and laboratory environments (Crabbe et al., 1999). These tests confirmed that PACAP KO mice show hyperactivity, elevated behavioral responses to novelty, and abnormal anxiety-like behavior, suggesting that these phenotypes are robust and reliable. Unlike the results in the studies discussed above, the mutant mice exhibited (1) increased social behaviors, (2) decreased startle response, (3) normal responses in the PPI test, and (4) slightly decreased depression-like behavior. Moreover, mild performance deficit in working memory was found to be associated with PACAP deficiency. These results suggest that PACAP has an important role in the regulation of locomotor activity, social behavior, anxiety-like behavior and, potentially, working memory.

## Materials and methods

### Animals and experimental design

Generation of PACAP KO mice by a gene-targeting technique using 129/Ola ES cell line has been reported previously (Hashimoto et al., 2001). Due to the high postnatal mortality rate in some genetic backgrounds, insufficiently backcrossed mice, or outbred mice were used in previous studies. Genetic background may have a profound influence on behavioral phenotypes (Crawley et al., 1997; Matsuo et al., 2010), and flanking genes might be responsible for the phenotypes observed in mutant mice (Gerlai, 1996; Silva et al., 1997; Crusio, 2004). Thus, there is a possibility that previously reported behavioral abnormalities were caused by the effects of polymorphisms in the genetic background or flanking genes on behavior rather than PACAP deletion, as only two or three backcrosses were conducted. To tightly control the genetic background, we prepared the two strains of heterozygous mice that were backcrossed at least 10 times with C57BL/6J or six times with 129SvEv. A congenic line is statistically expected to be 98.44% and 99.90% from the host after 6 and 10 generations of backcrossing, respectively (Rogner and Avner, 2003). We then obtained F1 homozygous PACAP KO mice (*n* = 20) and wild-type control littermates (*n* = 20) by mating heterozygous C57BL/6J and heterozygous 129SvEv mice. The genotypes of mice were identified by genomic PCR. Primers F322 (5′-ATCTCCTGTCATCTCACCTTGCTCCT-3′) and R812 (5′-GAAGAACTCGTCAAGAGAGGCGATAGAAG-3′) produced 491 bp PCR products from mutant tail DNA. Primers F382 (5′-ACCGAAAACAAATGGCTGTC-3′) and R670 (5′-GGTCCACAAAGTATATCTGTGCATTCTC-3′) amplified 293 bp PCR products from normal DNA. All behavioral tests were carried out with the F1 male mice that were at least 13 weeks old at the start of testing. Raw data of the behavioral test, the date on which each experiment was done, and the age of the mice at the time of the experiment are shown in the mouse phenotype database (Table 1; http://www.mouse-phenotype.org/). Mice were group housed (two wild-type mice and two mutant mice per cage) in a room with a 12 h light/dark cycle (lights on at 7:00 am) with access to food and water *ad libitum*. Room temperature was kept at 23 ± 2°C. Behavioral testing was performed between 9:00 am and 6:00 pm. After the tests, all apparatus was cleaned with diluted sodium hypochlorite solution to prevent a bias due to olfactory cues. To minimize the effects of previous tests on subsequent tests, we performed the behavioral test battery in a specific order, in which the less stressful tests preceded the more stressful tests. In this study, the tests were performed in the following sequence: neurological screens and wire hang (GHNS), LD, OF, EP, HP, one-chamber SI test, sociability and social novelty preference test, RR, startle response/PPI test, PS test, RM, TM, social interaction test in home cage (HC-SI), HC activity. Each behavioral test was separated from each other at least by one day.

**Table 1:** **Comprehensive behavioral test battery of PACAP KO mice**.

**Test**	**Age (w)**
1. Neurological screen	13–15
2. Light/dark transition	13–15
3. Open field	13–16
4. Elevated plus-maze	14–16
5. Hot plate	14–16
6. Social Interaction (novel environment)	14–16
7. Sociability/social novelty	14–17
8. Rotarod	15–17
9. Prepulse inhibition	15–17
10. Porsolt forced swim	15–18
11. Eight-arm radial maze	17–23
12.T-maze	21–26
13. Social interaction (home cage)	78–81

Age (w): age of weeks of subjects at the beginning of each test.

When using a behavioral test battery, the effects of the preceding test may confound the results obtained in the following tests. For example, the behavioral phenotype could be occluded or emerged in a test by the experiences and/or stresses experienced in prior tests. However, there are a few advantages to employing this testing strategy. First, it is an efficient tool for elucidating the functional significance of the gene of interest. By using such a test battery, a large amount of data can be obtained from a valuable set of mice. It is also useful for high-throughput screening of animal models of neuropsychiatric disorders (Powell and Miyakawa, 2006; Takao et al., 2007). Second, the behavioral phenotype of mice can be assessed with less stress, especially in the later stages of the test battery. As mice are subjected to various kinds of behavioral tests, they get used to being exposed to novel environments and researchers, which may be potential sources of stress. There is also a possibility that behavioral tests provide beneficial stimuli like an enriched environment. Finally, data acquired using the behavioral test battery may be useful for understanding the effects of experience × genotype interactions on behavioral abnormalities. So far, with the test battery, data has been collected from 140 genetically engineered strains of mice and their controls; some of these data are available in a public database (http://www.mouse-phenotype.org/). By utilizing the data derived from the test battery, it will be of interest to assess, using formally designed experiments, the potential influence of experience × genotype interactions on behavioral phenotype by comparing the results obtained from naïve mice with those from mice subjected to these tests. This would be particularly important for putative mouse models of neuropsychiatric disorders, considering the fact that gene × environment interactions play an essential role in the etiology of such disorders.

All behavioral testing procedures were approved by the Animal Research Committee, Graduate School of Medicine, Kyoto University.

### Behavioral tests

#### Neurological screen

The righting, whisker touch, and ear twitch reflexes were evaluated. A number of physical features, including the presence of whiskers or bald hair patches, were also recorded.

#### Hot plate test

The HP test was used to evaluate sensitivity to a painful stimulus. Mice were placed on a 55.0 (±0.3)°C HP (Columbus Instruments International, Columbus, OH), and latency to the first hind-paw response was recorded. The hind-paw response was defined as either a foot shake or a paw lick.

#### Rotarod test

Motor coordination and balance were tested with the RR test. The RR test, using an accelerating RR (UGO Basile North America Inc., Collegeville, PA), was performed by placing mice on rotating drums (3 cm diameter) and measuring the time each animal was able to maintain its balance on the rod. The speed of the RR accelerated from 4 to 40 rpm over a 5-min period. The animals went through three trials per day on two consecutive days. The trials were separated by more than 1 h intertrial intervals. It is possible to run a total of 40 mice consecutively in one trial before moving to the next one.

#### Startle response/PPI test

A startle reflex measurement system was used (O'HARA & CO., Tokyo, Japan). A test session began by placing a mouse in a Plexiglas cylinder where it was left undisturbed for 10 min. The duration of white noise that was used as the startle stimulus was 40 ms for all trial types. The startle response was recorded for 140 ms (measuring the response every 1 ms) starting with the onset of the prepulse stimulus. The background noise level in each chamber was 70 dB. The peak startle amplitude recorded during the 140-ms sampling window was used as the dependent variable. A test session consisted of six trial types (i.e., two types for startle stimulus-only trials, and four types for PPI trials). The intensity of the startle stimulus was 110 or 120 dB. The prepulse sound was presented 100 ms before the startle stimulus, and its intensity was 74 or 78 dB. Four combinations of prepulse and startle stimuli were employed (74–110 dB, 78–110 dB, 74–120 dB, and 78–120 dB). Six blocks of the six trial types were presented in a pseudorandom order such that each trial type was presented once within a block. The average inter-trial interval was 15 s (range, 10–20 s).

#### Open field test

Locomotor activity was measured using an OF test. Each mouse was placed in the corner of the OF apparatus (40 × 40 × 30 cm; Accuscan Instruments, Columbus, OH). The chamber of the test was illuminated at 100 lux. Total distance traveled, vertical activity (rearing measured by counting the number of photobeam interruptions), time spent in the center area (20 × 20 cm), and beam-break counts for stereotyped behaviors were recorded. If animals break the same beam (or set of beams) repeatedly, then the monitor considers that the animals are exhibiting stereotypy. This typically happens during grooming and head bobbing, for example. The stereotypy count is the number of beam breaks that occur during this period of stereotypic activity. Resting time is calculated as the difference between total time and time spent moving. Data were collected for 120 min.

#### Locomotor activity monitoring in the home cage

Locomotor activity monitoring in the HC was performed with PACAP KO mice. A system that automatically analyzes the locomotor activity of mice in their HC was used (Miyakawa et al., 2003). The system contains a HC (29 × 18 × 12 cm) and a filtered cage top, separated by a 13-cm-high metal stand containing an infrared video camera, which is attached to the top of the stand. Each mouse was individually housed in each HC, and their locomotor activity was monitored for six days. Only 16 mice out of 20 mice were used for each genotype due to the limited availability of apparatus. Data acquisition failed in one apparatus, which was measuring the activity of a single KO mouse. We analyzed the data for the remaining 16 WT and 15 KO mice. Distance traveled was measured automatically using Image HA software (see “Data analysis”).

#### Light/dark transition test

A LD test was conducted as previously described (Takao and Miyakawa, 2006b). The apparatus used for the LD test comprised a cage (21 × 42 × 25 cm) divided into two sections of equal size by a partition with a door (O'HARA & CO., Tokyo, Japan). One chamber was brightly illuminated (390 lux), whereas the other chamber was dark (2 lux). Mice were placed into the dark side and allowed to move freely between the two chambers with the door open for 10 min. The total number of transitions, latency to first enter the lit chamber, distance traveled, and time spent in each chamber were recorded by Image LD4 software (see “Data analysis”).

#### Elevated plus-maze test

An EP test was conducted as previously described (Komada et al., 2008). The EP consisted of two open arms (25 × 5 cm) and two enclosed arms of the same size with 15-cm high transparent walls. The arms and central square were made of white plastic plates and were elevated 55 cm above the floor. To minimize the likelihood of animals falling from the apparatus, 3-mm high Plexiglas walls surrounded the sides of the open arms. Arms of the same type were located opposite from each other (O'HARA & CO., Tokyo, Japan). Each mouse was placed in the central square of the maze (5 × 5 cm), facing one of the closed arms. Mouse behavior was recorded during a 10-min test period. The number of entries into an arm, and the time spent in the open and enclosed arms were recorded. Percentage of entries into open arms, time spent in open arms, number of total entries, and total distance traveled were analyzed. One of the wild-type mice fell from the apparatus and the recording had to be stopped. We excluded the data for this mouse. Data acquisition and analysis were performed automatically, using Image EP software (see “Data analysis”).

#### Porsolt forced swim test

The PS test apparatus consisted of four Plexiglas cylinders (20 cm high × 10 cm diameter). A non-transparent panel separated the cylinders to prevent the mice from seeing each other (O'HARA & CO., Tokyo, Japan). The cylinders were filled with water (23°C) up to a height of 7.5 cm. Mice were placed into the cylinders, and the immobility and the distance traveled were recorded over a 10-min test period. Images were captured at one frame per second. For each pair of successive frames, the amount of area (pixels) within which the mouse moved was measured. When the amount of area was below a certain threshold, mouse behavior was judged as “immobile.” When the amount of area equaled or exceeded the threshold, the mouse was considered as “moving.” The optimal threshold by which to judge was determined by adjusting it to the amount of immobility measured by human observation. Immobility lasting for less than a 2 s was not included in the analysis. Retention tests were administered 24 h after training. Data acquisition and analysis were performed automatically, using Image PS software (see “Data Analysis”).

#### Social interaction test in a novel environment

In the SI test, two mice of identical genotypes that were previously housed in different cages were placed in a box together (40 × 40 × 30 cm) and allowed to explore freely for 10 min (Tanda et al., 2009). Since a pair of mice was used as a sample in the test, the number of samples is half. Social behavior was monitored with a CCD camera connected to a Macintosh computer. Analysis was performed automatically using Image SI software (see “Data analysis”). The total number of contacts, total duration of active contacts, total contact duration, mean duration per contact, and total distance traveled were measured. The active contact was defined as follows. Images were captured at three frames per second, and distance traveled between two successive frames was calculated for each mouse. If the two mice contacted each other and the distance traveled by either mouse was longer than 4 cm, the behavior was considered as an “active contact.”

#### Social interaction test in home cage

Social interaction monitoring in the HC was conducted as previously described (Miyakawa et al., 2003). The system was same apparatus in locomotor activity in HC. Two mice of the same genotypes that had been housed separately were placed together in a HC. Their social behavior was then monitored for one week. Output from the video camera was fed into a Macintosh computer. Images from each cage were captured at a rate of one frame per second. Social interaction was measured by counting the number of particles detected in each frame: two particles indicated that the mice were not in contact with each other; and one particle (i.e., the tracking software could not distinguish two separate bodies) indicated contact between the two mice. We also measured locomotor activity during these experiments by quantifying the number of pixels that changed between each pair of successive frames. Only eight pairs of mice were used for each genotype due to the limited availability of apparatus. Data acquisition failed in one apparatus, which was measuring the activity of a pair of KO mice. Therefore, we analyzed the data for the remaining eight pairs of WT and seven pairs of KO mice. Analysis was performed automatically using Image HA software (see “Data analysis”).

#### Sociability and social novelty preference test

The test for sociability and preference for social novelty is well-designed method to investigate the complex genetics of social behaviors (Crawley, 2004; Moy et al., 2004). The apparatus comprised a rectangular, three-chambered box, and a lid containing an infrared video camera (O'HARA & CO., Tokyo, Japan). Each chamber was 20 × 40 × 22 cm and the dividing walls were made from clear Plexiglas, with small square openings (5 × 3 cm) allowing access into each chamber. We modified the method described by Moy et al. (2004) as follows: a habituation session was not performed in the apparatus, and the wire cages in the lateral compartments were located in a corner. An unfamiliar C57BL/6J male (stranger 1) that had no prior contact with the subject mouse was placed in one of the side chambers. The placement of stranger 1 in the left or right side chambers was systematically alternated between trials. The stranger mouse was enclosed in a small, circular wire cage, which allowed nose contact between the bars, but prevented fighting. The cage was 11 cm high, with a bottom diameter of 9 cm, and bars spaced 0.5 cm apart. The subject mouse was first placed in the middle chamber and allowed to explore the entire social test box for 10-min. The amount of time spent within a 5-cm distance of the wire cage in each chamber and in each chamber was measured with the aid of a camera fitted on top of the box. After the first 10 min, each mouse was tested in a second 10-min session to quantify social preference for a new stranger. A second, unfamiliar mouse was placed in the chamber that had been empty during the first 10-min session. This second stranger was enclosed in an identical small wire cage. The test mouse had a choice between the first, already-investigated unfamiliar mouse (stranger 1), and the novel unfamiliar mouse (stranger 2). As described above, the amount of time spent within a 5-cm distance of each wire cage and in each chamber during the second 10-min session was recorded. The stranger mice used in this experiment were 8–12-week-old C57BL/6J male mice, not littermates. Analysis was performed automatically using Image CSI software (see “Data analysis”).

#### Eight-arm radial maze

RM test was performed using fully-automated RM apparatuses (Yamasaki et al., 2008) (O'HARA & CO., Tokyo, Japan). The floor of the maze was made of white plastic, and the wall (25 cm high) consisted of transparent plastic. Each arm (9 × 40 cm) radiated from an octagonal central starting platform (perimeter 12 × 8 cm) like the spokes of a wheel. Identical food wells (1.4 cm deep and 1.4 cm in diameter) with pellet sensors were placed at the distal end of each arm. The pellets sensors were able to automatically record pellet intake by the mice. The maze was elevated 75 cm above the floor and placed in a dimly lit room with several extra-maze cues. During the experiment, the maze was maintained in a constant orientation. One week before pre-training, animals were deprived of food until their body weight was reduced to 80–85% of the initial level. In the pre-training, each mouse was placed in the central starting platform and allowed to explore and consume food pellets scattered on the whole maze for a 30-min period (one session per mouse). After completion of the initial pre-training, mice received another pre-training to take a food pellet from each food well after being placed at the distal end of each arm. A trial was finished after the mouse consumed the pellet. This was repeated eight times, using eight different arms, for each mouse. After these pre-training trials, actual maze acquisition trials were performed. In the spatial working memory task of the RM, all eight arms were baited with food pellets. Mice were placed on the central platform and allowed to obtain all eight pellets within 25 min. A trial was terminated immediately after all eight pellets were consumed or 25 min had elapsed. An “arm visit” was defined as traveling more than 5 cm from the central platform. The mice were confined at the center platform for 5 s after each arm choice. The animals went through one trial per day. For each trial, arm choice, latency to obtain all pellets, distance traveled, number of different arms chosen within the first eight choices, the number of arm revisited, and omission errors were automatically recorded. Data acquisition, control of guillotine doors, and data analysis were performed by Image RM software (see “Data analysis”). One of the KO mice died before the test and four mice died during the test. Therefore, we analyzed the data for the remaining 20 WT and 15 KO mice.

#### T-maze forced alternation task

The forced alternation task was conducted using an automatic TM that we devised (Takao et al., 2008; Shoji et al., 2012) (O'Hara & Co., Tokyo, Japan). It was constructed of white plastics runways with walls 25-cm high. The maze was partitioned off into six areas by sliding doors that can be opened downward. The stem of T was composed of area S2 (13 × 24 cm) and the arms of T were composed of area A1 and A2 (11.5 × 20.5 cm). Area P1 and P2 were the connecting passage way from the arm (area A1 or A2) to the start compartment (area S1). The end of each arm was equipped with a pellet dispenser that could provide food reward. The pellet sensors were able to record automatically pellet intake by the mice. Only eight WT mice and eight KO mice were subjected to the TM due to the availability of apparatus. One of KO mice escaped from the apparatus many times. We analyzed the data from the remaining seven KO mice. One week before the pre-training, mice were deprived of food until their body weight was reduced to 80–85% of the initial level. We provided daily eight sucrose pellets per mouse in addition to standard pellet chow to habituate to the sucrose pellets until beginning the pre-training. Mice were kept on a maintenance diet throughout the course of all the TM experiments. Before the pre-training, mice were subjected to 30-min adaptation session, during which they were allowed to freely explore the TM with all doors open and both arms baited with food. From one day after the adaptation session, mice received pre-training to take a food pellet from food tray. With all the doors closed and the pellet deposited in the food tray, mouse was placed into area A1. After the pellet was consumed or 5 min had elapsed, the mouse was trained likewise in area A2. Such training was repeated five times a day, and continued until the mice consumed more than 80% of the pellets. After the pre-training, mice were subjected to a forced alternation protocol for nine days. In the forced alternation task, each trial consisted of a forced-choice run followed by a free-choice run. A mouse was subjected to 10 consecutive trials in a session per day (cutoff time, 50 min). On a forced-choice run, the mouse was forced to choose one of the arms of the T (area A1 or A2), and received a sucrose pellet as a reward at the end of the arm. On a free-choice run, if the mouse entered the opposite arm that it was forced to choose in the forced-choice run, the response is considered to be “Correct” and the mouse receives a sucrose pellet as a reward. If the mouse went to the same arm as that visited in the forced-choice run, the mouse was confined within the area for 10 s as a penalty (“Error” response). The mouse was given delay time for 3 s between the forced- and free-choice runs. After the mouse consumed the pellet or the mouse stayed more than 10 s without consuming the pellet, door that separated the arm (area A1 or A2) and connecting passage way (area P1 or P2) would be opened and the mouse could return to the starting compartment (area S1), via connecting passage way, by itself. The arm chosen in the forced-choice run was varied pseudo-randomly across trials using Gellermann schedule so that mice received equal numbers of left and right presentations. A variety of fixed extra-maze clues surrounded the apparatus. On the 10th day, delay (10, 30, or 60 s) was applied after the forced-choice run. Data acquisition, control of sliding doors, and data analysis were performed by Image TM software (see “Data analysis”).

#### T-maze left-right discrimination task

In the left-right discrimination task, each mouse was given a free-choice run of 10 trials in a session (cutoff time, 50 min). A sucrose pellet was always delivered to the food tray of one of the arms, namely, the goal arm. The location of the goal arm was invariable across trials and sessions, and was counterbalanced across control and PACAP KO mice. When the mouse entered the goal arm, it was considered a correct response. If the mouse ate the pellet or 10 s elapse, passage way (area P1 or P2) was opened and the mouse could return to the starting compartment (area S1), via connecting passage way, by itself. Mice were subjected to a left-right discrimination protocol for five days. On the 6th and 10th day, we started additional sessions to the mice to assess behavioral flexibility by placing the reward in the opposite, previously unbaited arm (i.e., reversal learning). Data acquisition, control of sliding doors, and data analysis were performed by Image TM software (see “Data analysis”).

### Data analysis

Behavioral data were obtained automatically by applications based on the public domain NIH Image program and Image J program and modified for each test by Tsuyoshi Miyakawa (available through O'HARA & CO., Tokyo, Japan). Statistical analysis was conducted using StatView (SAS Institute, Cary, NC). Data were analyzed using a paired *t*-test, one-way ANOVA, or two-way repeated measures ANOVA. Since our behavior test battery included 14 tests and 75 indices for statistical analysis, we also re-evaluated our data be conservative Bonferroni correction. Values in graphs are expressed as mean ± SEM.

## Results

### Increased locomotor activity in PACAP KO mice

To evaluate the behavioral effects of PACAP deficiency, we subjected PACAP KO mice and their wild-type littermates to a comprehensive battery of behavioral tests. PACAP KO mice showed no obvious differences in their physical characteristics. There were no significant differences in body weight (*F*_(1, 38)_ = 0.055, *p* = 0.8166), body temperature (*F*_(1, 38)_ = 0.64, *p* = 0.4286), neuromuscular strength (as assessed by wire hang and grip strength tests, *F*_(1, 38)_ = 0.845, *p* = 0.3638, and *F*_(1, 38)_ = 0.159, *p* = 0.6924, respectively), or sensitivity to a painful stimulus (HP test, *F*_(1, 38)_ = 0.618, *p* = 0.4365) between the wild-type and KO mice. Raw data and summary data (mean ± SEM) of these behavioral tests are shown in the mouse phenotype database (http://www.mouse-phenotype.org/).

**Figure 1:**
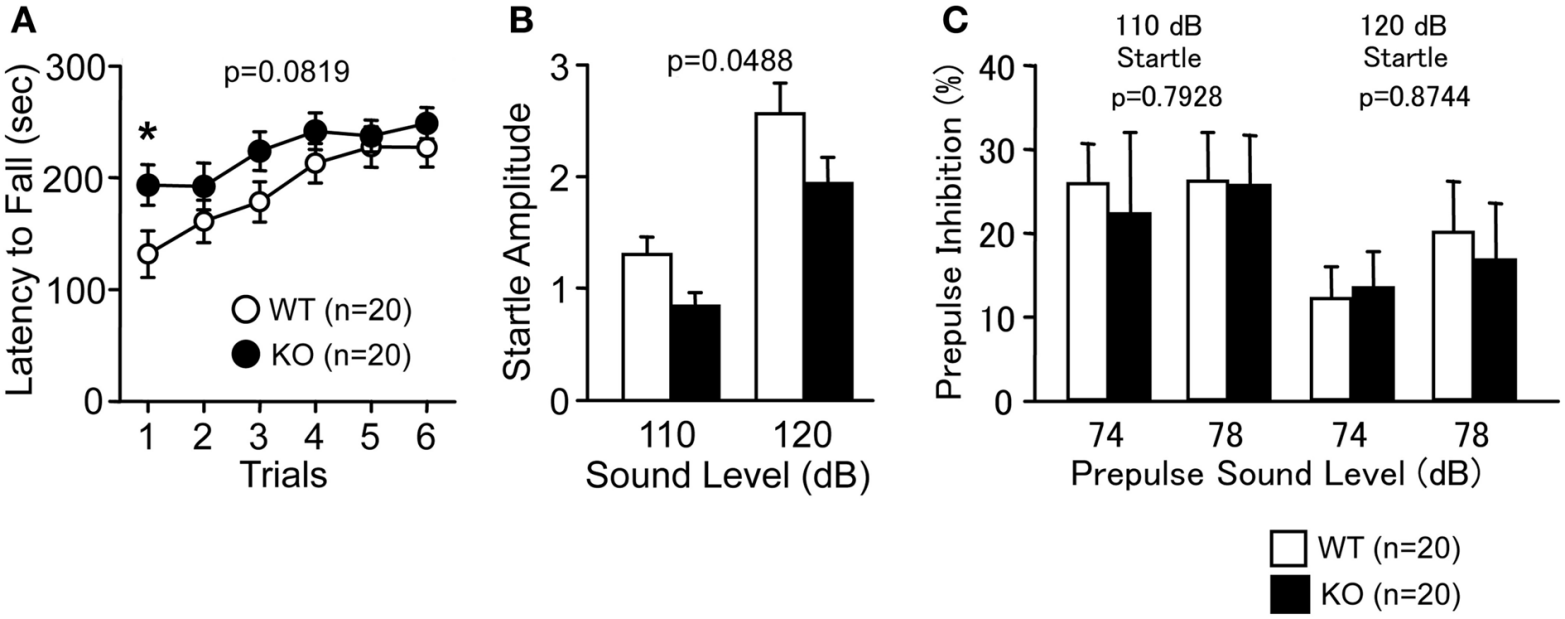
**Motor coordination and startle response/prepulse inhibition in PACAP KO mice and wild-type mice. (A)** Latency to fall from the rotating drum was shown in the rotarod test. Acoustic startle response **(B)** and prepulse inhibition **(C)** were shown. Data are presented as means ± SEM for the indicated numbers of animals. The *p*-values indicate genotype effect in two-way repeated measures ANOVA.

Latency to fall off the RR tended to be longer in PACAP KO mice compared with wild-type mice (Figure 1A; genotype effect, *F*_(1, 38)_ = 3.194, *p* = 0.0819; genotype × trial interaction effect, *F*_(5, 190)_ = 0.952, *p* = 0.4485). Indeed, PACAP KO mice showed significantly longer latency than controls in the first trial (*F*_(1, 38)_ = 4.954, *p* = 0.032). PACAP KO mice also exhibited a significantly decreased acoustic startle response compared with wild-type mice (Figure 1B; *F*_(1, 38)_ = 4.145, *p* = 0.0488), however, PPI was not significantly different across genotypes (Figure 1C; *F*_(1, 38)_ = 0.07, *p* = 0.7928 (110 dB); *F*_(1, 38)_ = 0.025, *p* = 0.8744 (120 dB)).

**Figure 2:**
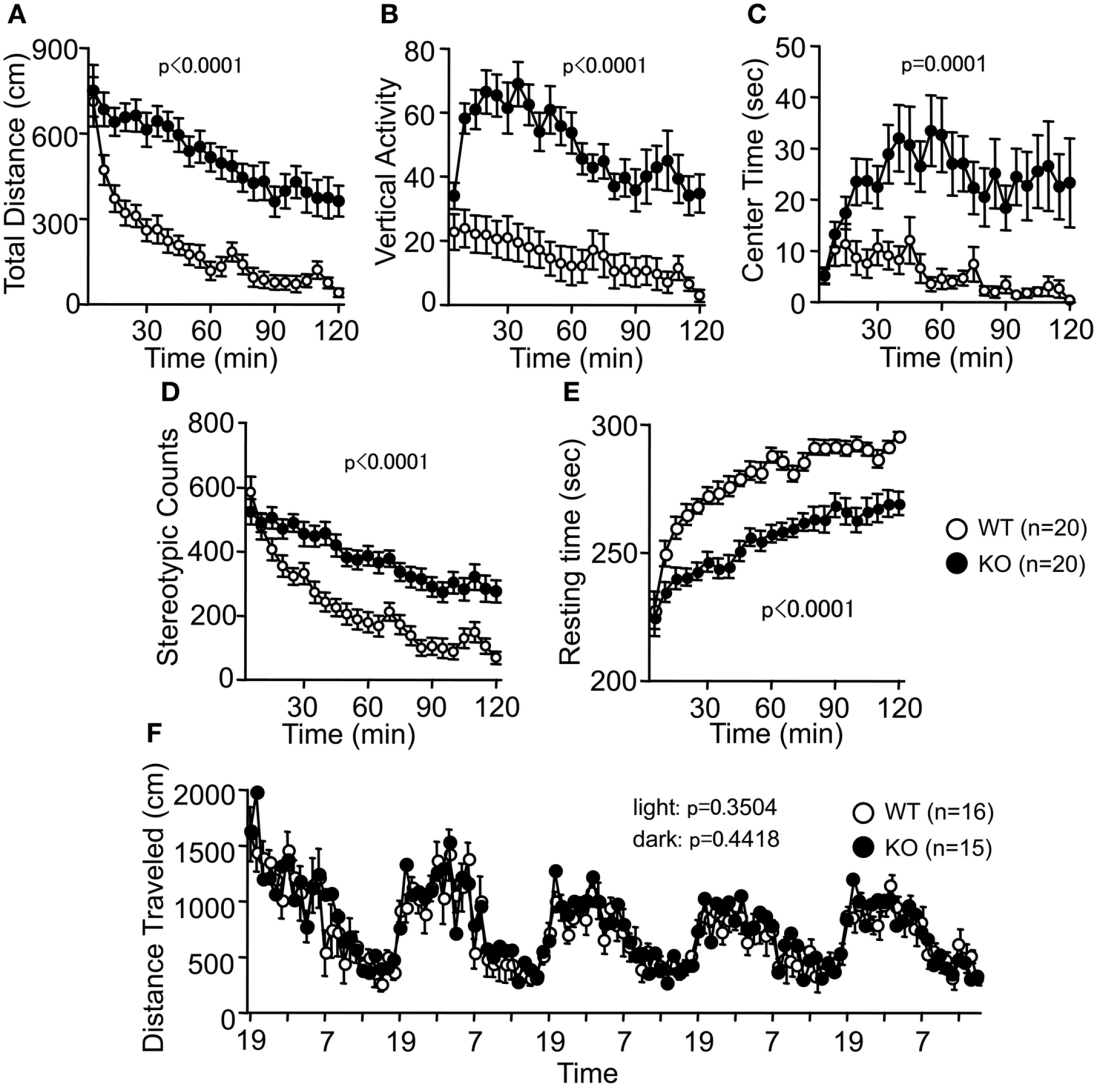
**Increased locomotor activity of PACAP KO mice in the open field test. (A)** Distance traveled in the open field test was significantly increased in the PAPAC KO mice compared with wild-type mice. Counts of vertical activity **(B)**, time spent in the center of the compartment **(C)**, counts of stereotypic behavior **(D)**, and resting time **(E)** were shown. **(F)** There were no significant differences in the distance traveled in the home cage between WT and KO mice. Data are presented as means ± SEM for the indicated numbers of animals. The *p*-values indicate genotype effect in two-way repeated measures ANOVA.

**Figure 3:**
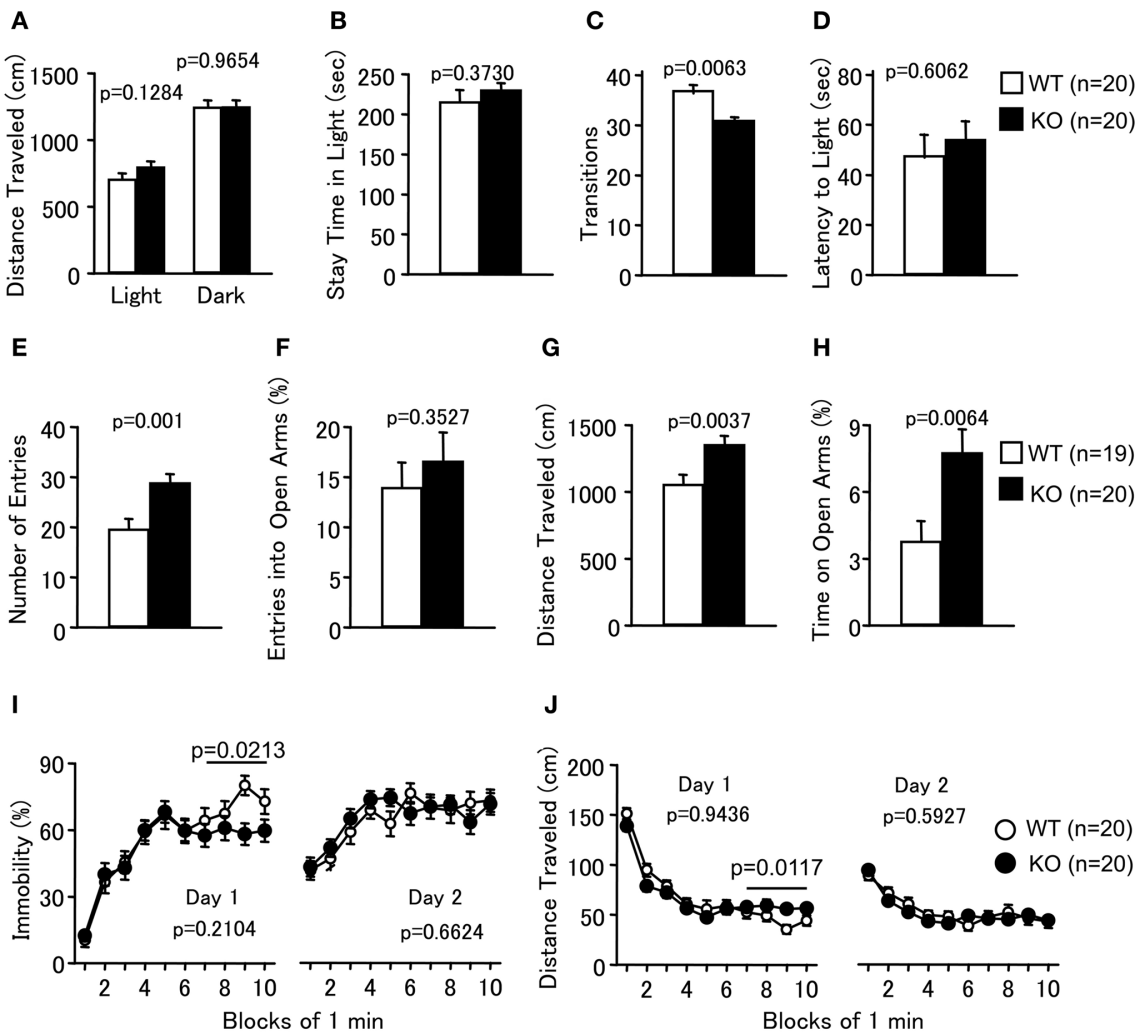
**Abnormal anxiety-like and slightly decreased depression-like behaviors in PACAP knockout mice. (A–D)** Light/dark transition test: the distance traveled in the light/dark compartments **(A)**, time spent in the light compartment **(B)**, number of light/dark transitions **(C)**, and latency to enter the light compartment **(D)** were shown. **(E–H)** Elevated plus maze: the number of arm entries **(E)**, percentage of entries into the open arms **(F)**, distance traveled **(G)**, and percentage of time spent on the open arms **(H)** were shown. **(I,J)** Porsolt forced swim test: the percentage of immobility time for day 1 and day 2 **(I)** and distance traveled for day 1 and day 2 **(J)** were shown. Data are presented as means ± SEM for the indicated numbers of animals. The *p*-values indicate the genotype effects in one-way ANOVA **(A–H)** and two-way repeated measures ANOVA **(I,J)**.

**Figure 4:**
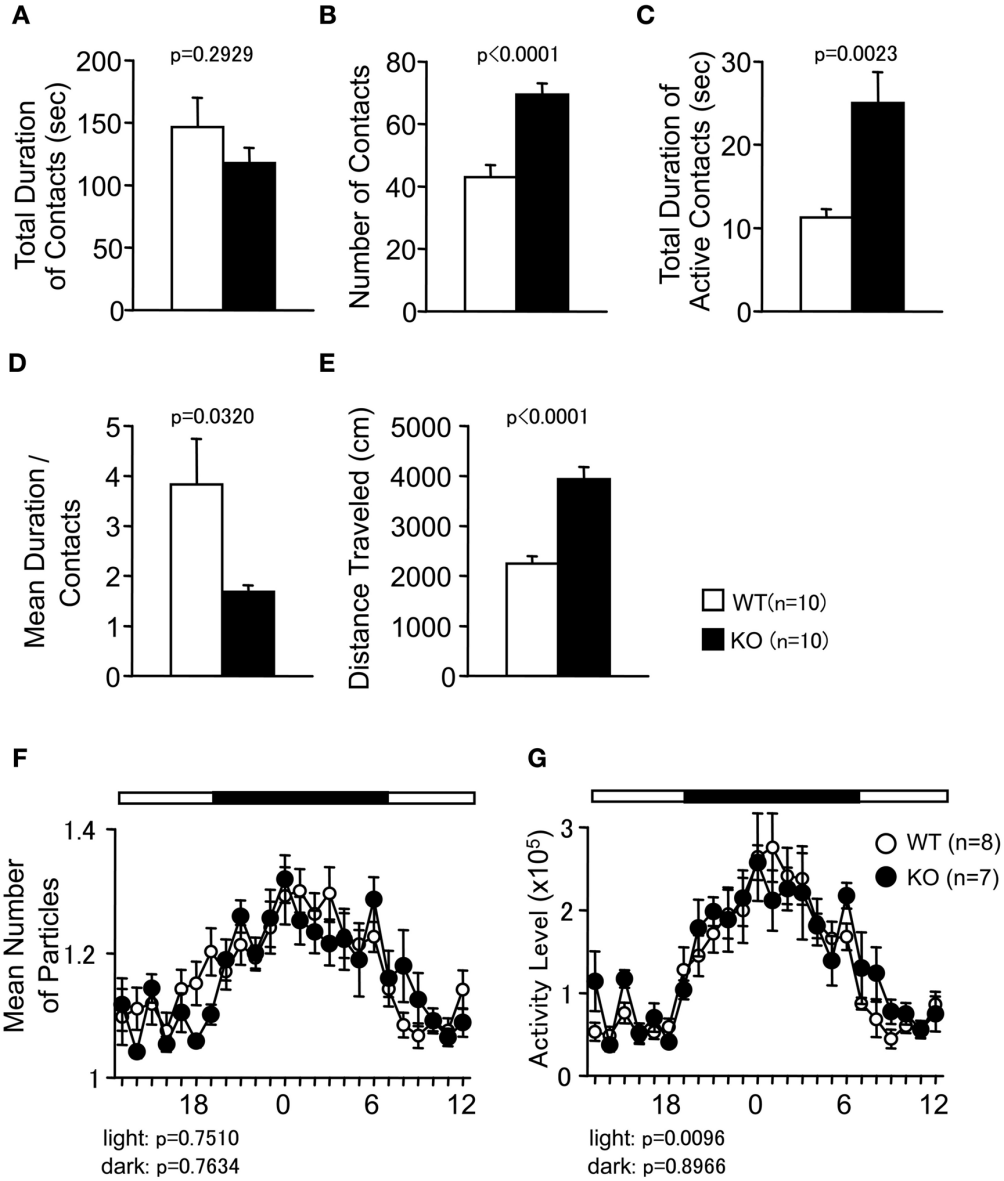
**Social behaviors of PACAP KO mice in a novel environment and home cage**. **(A–E)** Social interaction in a novel environment: the total duration of contacts **(A)**, number of contacts **(B)**, total duration of active contacts **(C)**, mean duration of each contact **(D)**, and total distance traveled **(E)** were shown. **(F,G)** Social interaction in home cage: the mean number of particle detected **(F)** and activity level **(G)** over six days were shown. Data are presented as means ± SEM of three days for the indicated numbers of animals. The *p*-values indicate the genotype effect in one-way ANOVA **(A–E)** and two-way repeated measures ANOVA **(F,G)**.

Spontaneous locomotor activity was tested in the OF test. Consistent with previous reports (Hashimoto et al., 2001; Tanaka et al., 2006), there were significant differences between genotypes in the total distance traveled (Figure 2A; *F*_(1, 38)_ = 31.146, *p* < 0.0001). PACAP KO mice also exhibited increased vertical activity (Figure 2B; *F*_(1, 38)_ = 30.061, *p* < 0.0001), time spent in the center area (Figure 2C; *F*_(1, 38)_ = 18.128, *p* = 0.0001), stereotypic behaviors (Figure 2D; *F*_(1, 38)_ = 24.543, *p* < 0.0001), and reduced resting time (Figure 2E; *F*_(1, 38)_ = 33.741, *p* < 0.0001). Increased locomotor activity of PACAP KO mice was consistently detected in several behavioral tasks. For example, total distance traveled by PACAP KO mice was significantly greater than that traveled by controls during the EP test (Figure 3G; *F*_(1, 37)_ = 9.585, *p* = 0.0037), the SI test in a novel environment (Figure 4E; *F*_(1, 18)_ = 34.89, *p* < 0.0001), and sociability and social novelty preference tests (data not shown; *F*_(1, 37)_ = 26.643, *p* < 0.0001, and *F*_(1, 37)_ = 67.103, *p* < 0.0001, respectively). However, no significant difference was detected in the distance traveled in the HC between the mutant and wild-type mice (Figure 2F; dark period, *F*_(1, 29)_ = 0.608, *p* = 0.4418; light period, *F*_(1, 13)_ = 0.901, *p* = 0.3504). These results therefore revealed a hyperlocomotor activity phenotype of PACAP KO mice in novel environments.

### Abnormal anxiety-like and slightly decreased depression-like behaviors in PACAP KO mice

In the LD test, PACAP KO mice showed no obvious differences between genotypes in the distance traveled (Figure 3A; light, *F*_(1, 38)_ = 2.416, *p* = 0.1284; dark, *F*_(1, 38)_ = 0.002, *p* = 0.9654), time spent in the light chamber (Figure 3B; *F*_(1, 38)_ = 0.813, *p* = 0.373), or first latency to enter the light chamber (Figure 3D; *F*_(1, 38)_ = 0.270, *p* = 0.6062). However, mutant mice exhibited a decrease in the number of transitions between chambers (Figure 3C; *F*_(1, 38)_ = 8.362, *p* = 0.0063). In contrast, the time spent in the central area of the OF (Figure 2C), the number of entries into the arms (Figure 3E; *F*_(1, 37)_ = 12.753, *p* = 0.001), and the percentage of time spent on the open arms (Figure 3H; *F*_(1, 37)_ = 8.37, *p* = 0.0064) of the EP were significantly greater for the PACAP KO mice compared with the wild-type mice, consistent with a previous report (Hashimoto et al., 2001). The percentage of entries into the open arms did not differ significantly between the wild-type and PACAP KO mice (Figure 3F; *F*_(1, 37)_ = 0.886, *p* = 0.3527). Increased stay time in open arms in the EP test and increased center stay time in OF test are generally considered as an indication of decreased anxiety. However, it would be also possible to interpret the abnormalities of the PACAP KO mice as increased anxiety-like behavior. PACAP KO mice showed a decrease in the number of transitions between chambers in the LD test, which is a well-validated index of anxiety-like behavior (Crawley, 1985). In addition, increased exploration of the open area in the EP could potentially reflect an increased panic-like escape response to stress (Holmes et al., 2000). The pattern of behavior shown by PACAP KO mice in the anxiety tests is similar to that of conditional calcineurin KO mice (Miyakawa et al., 2003), which was interpreted as an indication of enhanced anxiety-like behavior. Further study will be needed to clarify the role of PACAP in the regulation of anxiety.

Depression-like behavior of PACAP KO mice was next examined in the PS test, which measures the time of immobility in a small pool that contains no means of escape. We performed a separate analysis of the results obtained during the last 4 min of the test on Day 1 because the genotype × time interaction is a significant factor in the distance traveled (Figure 3J; genotype × time effect, *F*_(9, 342)_ = 2.509, *p* = 0.0086). PACAP KO mice showed significantly less immobility than wild-type mice (Figure 3I; total, genotype effect, *F*_(1, 38)_ = 1.623, *p* = 0.2104; genotype × time effect, *F*_(9, 342)_ = 1.518, *p* = 0.14; 7–10 min, genotype effect, *F*_(1, 38)_ = 5.765, *p* = 0.0213). They also demonstrated increased distance traveled relative to wild-type mice in the last 4 min at day 1 (Figure 3J; total, genotype effect, *F*_(1, 38)_ = 0.005, *p* = 0.9436; genotype × time effect, *F*_(9, 342)_ = 2.509, *p* = 0.0086; 7–10 min, genotype effect, *F*_(1, 38)_ = 7.009, *p* = 0.0117). However, there were no significant differences in immobility at day 2 between the mutant and wild-type mice (Figure 3I; genotype effect, *F*_(1, 38)_ = 0.194, *p* = 0.6624; genotype × time effect, *F*_(9, 342)_ = 1.003, *p* = 0.4376). In addition, no significant differences were detected in distance traveled at day 2 (Figure 3J; genotype effect, *F*_(1, 38)_ = 0.291, *p* = 0.5927; genotype × time effect, *F*_(9, 342)_ = 0.916, *p* = 0.5112). These observations indicate that PACAP deficiency might decrease depression-like behavior, although the effect was subtle.

### Increased social interaction in PACAP KO mice in sociability and social novelty preference test

In the SI test conducted in a novel environment, the number of contacts and total duration of active contacts between the animals were significantly greater in the PACAP KO mice compared with the wild-type mice (Figures 4B,C; *F*_(1, 18)_ = 24.862, *p* < 0.0001, and *F*_(1, 18)_ = 12.599, *p* = 0.0023, respectively). However, the total duration of contacts did not differ between genotypes (Figure 4A; *F*_(1, 18)_ = 1.174, *p* = 0.2929). Mean duration per contact was significantly shorter in PACAP KO mice than in the wild-type mice (Figure 4D; *F*_(1, 18)_ = 5.403, *p* = 0.0320). It is possible that these results were attributable to increased locomotor activity of the PAPAC KO mice in this test (Figure 4E; *F*_(1, 18)_ = 34.89, *p* < 0.0001).

To examine whether the increase in SI was the result of hyperactivity in the PACAP KO mice, the SI in the HC was also monitored under familiar conditions over a 6-day period and in sociability and social novelty preference test. In the SI test in the HC, time spent separated is usually increased when mice are active and decreased when mice are sleeping. There were no significant differences between genotypes in the mean numbers of particles detected (Figure 4F; dark period, *F*_(1, 13)_ = 0.094, *p* = 0.7634; light period, *F*_(1, 13)_ = 0.105, *p* = 0.7510). Compared with wild-type mice, PACAP KO mice showed significantly greater locomotor activity as measured by the number of pixels that changed between each pair of successive flames only during the light period in the HC (Figure 4G; dark period, *F*_(1, 13)_ = 0.018, *p* = 0.8966; light period, *F*_(1, 13)_ = 9.2, *p* = 0.0096).

**Figure 5:**
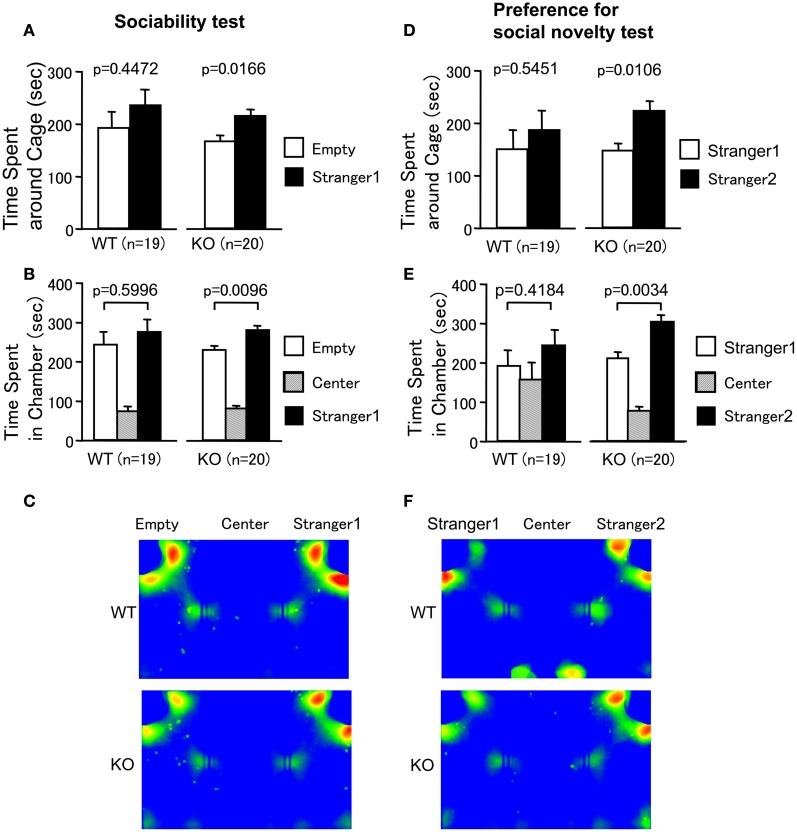
**Increased social behavior of PACAP KO mice in sociability and social novelty preference test**. **(A–C)** Sociability test: the time spent in the vicinity of the empty cage versus the cage containing a stranger (stranger 1) **(A)**, and the time spent in the chamber with the empty cage, with the cage containing a stranger (stranger 1), and without the cage (center) **(B)** were shown. **(C)** All traces of each mouse were superimposed and averaged images for the traces of WT mice (upper **C**) and PACAP KO mice (lower **C**) in sociability test are shown. **(D–F)** Preference for social novelty test: the time spent in the vicinity of the cage containing a stranger (stranger 2) and the cage containing a familiar (stranger 1) **(D)**, and the time spent in the chamber with the cage containing a stranger (stranger 2), with the cage containing a familiar (stranger 1), or without the cage (center) **(E)** were shown. **(F)** All traces of each mouse were superimposed and averaged images for the traces of WT mice (upper **F**) and PACAP KO mice (lower **F**) in preference for social novelty test are shown. Data are presented as means ± SEM for the indicated numbers of animals. The *p*-values indicate the genotype effect in the paired *t*-test.

Sociability and social novelty preference test consists of a sociability test and a social novelty preference test. This test assesses social interaction that is relatively independent of locomotor activity compared to the other SI test, because the preference of the mice can be quantified based on the time spent around a wire cage containing a stranger mouse versus the time spent around an empty cage in the sociability test. The preference for a stranger mouse versus a familiar mouse is tested in the social novelty preference test (Moy et al., 2004). In the sociability test, PACAP KO mice spent more time around the cage with the stranger than the empty cage (Figures 5A,C; wild-type mice, *t* = 0.777, *df* = 18, *p* = 0.4472; PACAP KO mice, *t* = 2.626, *df* = 19, *p* = 0.0166, stranger 1 side versus empty cage side). PACAP KO mice also showed a preference for the chamber with the stranger relative to the empty chamber (Figure 5B; wild-type mice, *t* = 0.534, *df* = 18, *p* = 0.5996; PACAP KO mice, *t* = 2.878, *df* = 19, *p* = 0.0096, stranger 1 side versus empty cage side).

In the social novelty preference test, social behavior was evaluated by contact with the first, already-investigated mouse (stranger 1) and the novel mouse (stranger 2) in the wire cage. PACAP KO mice demonstrated a preference for novelty (Figures 5D,F; wild-type mice, *t* = 0.617, *df* = 18, *p* = 0.5451; PACAP KO mice, *t* = 2.836, *df* = 19, *p* = 0.0106, stranger 1 side versus stranger 2 side). Consistently, PACAP KO mice also spent more time in the chamber with stranger 2 (Figure 5E; wild-type mice, *t* = 0.828, *df* = 18, *p* = 0.4184; PACAP KO mice, *t* = 3.3343, *df* = 19, *p* = 0.0034, stranger 1 side versus stranger 2 side). PACAP KO mice traveled a greater distance in both tests (data not shown; *F*_(1, 37)_ = 26.643, *p* < 0.0001 (sociability test); *F*_(1, 37)_ = 67.103, *p* < 0.0001 (social novelty preference test)). However, there were no significant differences in the number of entries into each cage and chamber of wild-type mice (data not shown; sociability test (stranger 1 side versus empty cage side); the number of entries into cages; *t* = 0.535, *df* = 18, *p* = 0.5993; the number of entries into chambers; *t* = 1.278, *df* = 18, *p* = 0.2175; social novelty preference test (stranger 1 side versus stranger 2 side); the number of entries into cages; *t* = 0.131, *df* = 18, *p* = 0.8975; the number of entries into chambers; *t* = 0.315, *df* = 18, *p* = 0.756) and those of PACAP KO mice (data not shown; sociability test (stranger 1 side versus empty cage side); the number of entries into cages; *t* = 0.046, *df* = 19, *p* = 0.9635; the number of entries into chambers; *t* = 0.566, *df* = 19, *p* = 0.5779; social novelty preference test (stranger 1 side versus stranger 2 side); the number of entries into chambers; *t* = 0.209, *df* = 19, *p* = 0.837), with the exception in the number of entries into cages in social novelty preference test (*t* = 2.464, *df* = 19, *p* = 0.0234). In our study, the stay time around a stranger mouse was similar to that around an empty one in wild-type mice (Figures 5C,F). The reason that the significant preference for stranger mouse was not seen in the wild-type mice is not known. The time spent around the cage might be determined by approach-avoidance conflict and our protocol that omitted habituation trial could have shifted the conflict toward avoidance behavior. In any case, we are interested in the genotype differences that affect this conflict. The data demonstrate that approach-avoidance conflict in PACAP KO mice shifts toward approaching behavior compared with wild-type mice. These results suggest that the increased social behavior of PACAP KO mice was not simply a result of their hyperlocomotor activity.

### Working memory performance of PACAP KO mice

**Figure 6:**
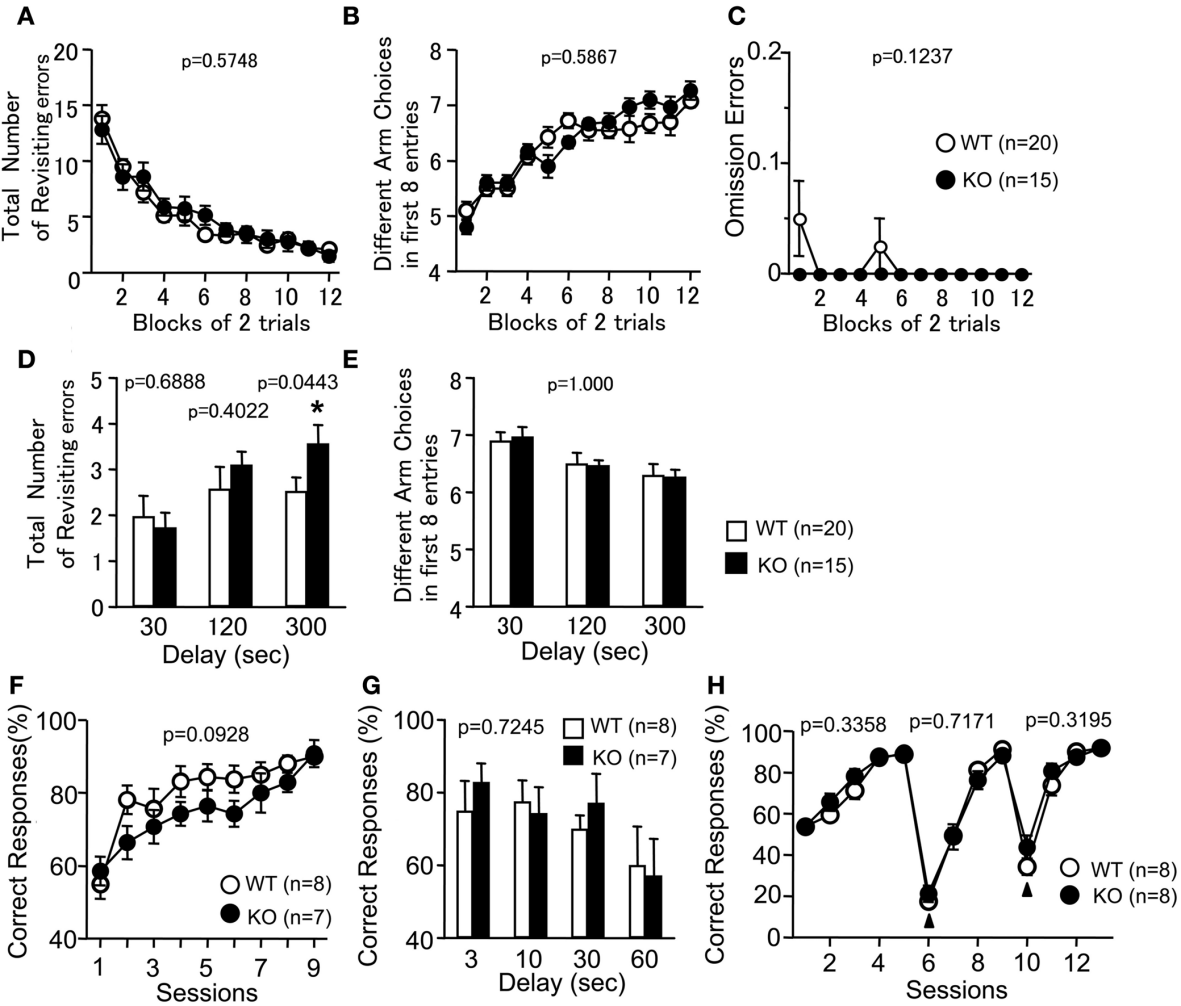
**Working memory performance of PACAP KO mice**. Radial Maze: Total number of arms revisited **(A,D)**, different arm choices among the first eight entries **(B,E)**, and omission errors **(C)** during training were shown. During trials 25–30, a delay was applied after the first four pellets were consumed **(D,E)**. Data are presented as means of two trials. **(F,G)** Forced alternation task: the percentage of correct choices was shown. During session 10, delay (3, 10, 30, 60 s) was applied between forced-choice and free-choice runs **(G)**. **(H)** Left-right discrimination task: the percentage of correct choices was shown. Arrowheads indicate the session placing the reward in the opposite arm. Data are presented as means ± SEM for the indicated numbers of animals. The *p*-values indicate genotype effect in two-way repeated measures ANOVA **(A–C, E–H)** and one-way ANOVA **(D)**.

To examine whether the loss of PACAP was associated with cognitive dysfunctions, PACAP KO mice were analyzed in RM and TM tests. In the RM test, the number of revisiting errors, in which subjects returned to the arms that had been visited previously to retrieve a food pellet, was not significantly different between genotypes during trials (Figure 6A; *F*_(1, 33)_ = 0.321, *p* = 0.5748). In the more difficult task with a delay period (300 s), the number of revisiting errors was increased in PACAP KO mice compared with wild-type mice (Figure 6D; with delays: 30 s, *F*_(1, 33)_ = 0.163, *p* = 0.6888; 120 s, *F*_(1, 33)_ = 0.72, *p* = 0.4022; 300 s, *F*_(1, 33)_ = 4.374, *p* = 0.0443). The number of different arm choices among the first eight entries, which is considered a task of working memory that is relatively independent of locomotor activity, as well as the total number of arm choices were not significantly different between genotypes (Figure 6B; without a delay, *F*_(1, 33)_ = 0.301, *p* = 0.5867; Figure 6E; with delays, *F*_(1, 33)_ = 0, *p* = 1.000). It demonstrates that reward consumption, as assessed by the number of omission errors, did not change throughout the test period (Figure 6C; *F*_(1, 33)_ = 2.496, *p* = 0.1237). This result suggests that reward value was not significantly different between genotypes during the test. It should be noted that four KO mice, but no wild-type mice, died during the RM test. Therefore, we cannot exclude the possibility that KO mice are more susceptible to the effects of dietary restriction, and that this potential phenotype served as an indirect cause of the performance deficit in the test.

To further assess the cognitive functions of the PACAP KO mice, the animals were subjected to a forced alternation task and a left-right discrimination task in the TM test. The forced alternation task is considered to provide a measure of working memory (Hepler et al., 1985; Mastropaolo et al., 1988). Correct responses in the forced alternation task tended to decrease in PACAP KO mice compared with wild-type mice (Figure 6F; session 1–9; genotype effect, *F*_(1, 13)_ = 3.292, *p* = 0.0928; genotype × time effect, *F*_(8, 104)_ = 1.006, *p* = 0.4362). To increase the difficulty of the task, a delay period (3, 10, 30, 60 s) was applied. Under these conditions, PACAP KO mice did not show significant abnormality in the percentage of correct choices (Figure 6G; *F*_(1, 13)_ = 0.013, *p* = 0.7245). In the left-right discrimination task, the percentage of correct choices did not differ between wild-type and PACAP KO littermates (Figure 6H; *F*_(1, 14)_ = 0.994, *p* = 0.3358). As shown in the results of the reversal-learning sessions (from session 6 to 9 and 10 to 13), PACAP KO mice also did not exhibit significant differences in correct responses (Figure 6H; session 6–9, *F*_(1, 14)_ = 0.137, *p* = 0.7171; session 10–13, *F*_(1, 14)_ = 1.065, *p* = 0.3195). These results demonstrate that PACAP KO mice showed mild performance deficit in working memory in both the RM and TM tests, and that they did not exhibit significant abnormality in reference memory and behavioral flexibility.

### The power of statistical analysis for the behavioral test battery

Some statistically significant behavioral abnormalities could be due to false-positives caused by the multiple statistical tests that are necessary when a number of behavioral tests are conducted. Since our behavior test battery included 14 tests and 75 indices for statistical analysis, we re-evaluated our data by conservative Bonferroni correction (the adjusted *P*-value at the 0.05 significance level for 75 indices is 0.00067). After conservative Bonferroni correction, results remained significant for total distance (*p* < 0.0001), vertical activity (*p* < 0.0001), center time (*p* = 0.0001), stereotypic counts (*p* < 0.0001) in OF test, number of contacts (*p* < 0.0001), and distance traveled (*p* < 0.0001) in SI test, demonstrating that PACAP regulates locomotor activity. By contrast, statistical significances disappeared in some indices when this conservative criterion was used. Future studies will be necessary to confirm the potential behavioral phenotypes that did not survive the Bonferroni correction, and that have not been duplicated in two or more independent studies.

## Discussion

The present study used a comprehensive battery of behavioral tests to analyze KO mice with a genetic disruption of PACAP. The lack of PACAP did not lead to significant abnormalities in overall health and appearance; however, PACAP KO mice demonstrated some significantly abnormal behaviors. We summarized the behavioral phenotypes in PACAP KO and PAC1 KO mice with different genetic backgrounds (Table 2). Increased locomotor activity and abnormal anxiety-like behavior, both of which were observed in previous studies (Hashimoto et al., 2001, 2007; Tanaka et al., 2006; Ishihama et al., 2010; Gaszner et al., 2012), were confirmed in the present study and can be considered robust and reliable phenotypes. On the other hand, our results were inconsistent with previous studies (Tanaka et al., 2006; Hashimoto et al., 2007, 2009; Ishihama et al., 2010; Gaszner et al., 2012) with regard to social behavior, startle response, PPI, and depression-like behavior.

**Table 2:** **Behavioral phenotypes in PACAP KO and PAC1 KO mice**.

**Strain**	**PACAP KO**	**PACAP KO**	**PACAP KO**	**PAC1 KO**	**PAC1 KO**	**PAC1 KO**	**Forebrain-specific PAC1 KO**
ES cell line	E14tg2a (129/Ola)	E14tg2a (129/Ola)	E14tg2a (129/Ola)	H1 (129/Sv)	H1 (129/Sv)	ET14/1 (129/Ola)	ET14/1 (129/Ola)
Donor strain	C57BL/6	C57BL/6	C57BL/6	C57BL/6J	C57BL/6J	C57BL/6	C57BL/6
Back ground	F1 homozygous mice obtained from intercross of heterozygous mice (C57BL/6J × 129SvEv)	F2- and F3-crossbred mice	CD1 (ICR)	F1–F4-crossbred strain	F2′ mice obtained from intercross of F1′ heterozygous mice (F4 KO × wild-type mice); offsprings obtained from hetrozygous female (F1′) × homozygous male (F2′)	75% C57BL/6/25% 129 Ola	75% C57BL/6/25% 129 Ola
Locomotor activity	Increased in a novel environment, but not in a home cage	Increased (but decreased with 0.2 mg/kg haloperidol)	Increased (but decreased with 2.0 mg/kg amphetamine, 0.1 mg/kg risperidone, or EE)	N/D	N/D	Increased	Normal
Anxiety-like behavior	Abnormal (decreased transitions in LD; increased time spent in open arm in EP)	Decreased (increased time spent in open arms and open arm entries in EP)	Decreased (decreased number of entries into the dark compartment and peeks into the light-box in LD; decreased number of marbles buried in the marble burying test)	N/D	N/D	Decreased (increased time spent and entries in open compartment and decreased latency to enter the compartment in EZ; increased time spent and vist in open arms in EP)	Normal
Social behavior	Increased contacts in social interaction test and sociability and social novelty preference test, but not in a home cage	N/D	Decreased duration of social interaction in the social interaction test (improved by EE)	N/D	Decreased social investigation after social cues or OVX female urine; excessive sexual mounting; reduced aggression	N/D	N/D
Startle response	Decreased	N/D	N/D	N/D	N/D	N/D	N/D
Prepulse inhibition	Normal	N/D	Impairment in PPI (reversed with 2.0 mg/kg amphetamine or 0.1 mg/kg risperidone)	N/D	N/D	N/D	N/D
Depression-like behavior	Slightly decreased immobility in PS	N/D	Increased immobility in the PS (decreased with 10–60 mg/kg desipramine, 0.1 mg/kg ritanserin, or EE)	N/D	N/D	N/D	N/D
Working memory	Mild performance deficit	N/D	N/D	N/D	N/D	N/D	N/D
Other behavioral phenotypes	Better performance in rotarod test	Increased exploratory behavior in the emergence test and novel-object test; explosive jumping behavior	Increased jumping behavior (reduced by EE)	Subtle memory impairment in contextual fear conditioning paradigms; shortened circadian period length	–	Memory impairment in contextual fear conditioning paradigms; normal circadian rhythm	Memory impairment in contextual fear conditioning paradigms; normal circadian rhythm
References	Present study	Hashimoto et al., 2001	Hashimoto et al., 2001, 2007, 2009; Tanaka et al., 2006; Ishihama et al., 2010; Gaszner et al., 2012	Sauvage et al., 2000; Hannibal et al., 2001	Nicot et al., 2004	Otto et al., 2001a,b	Otto et al., 2001a,b

The abbreviations used are: N/D, not determined; LD, light-dark transition test or light-dark box test; EP, elevated plus-maze test; EZ, elevated zero maze; PS, Porsolt forced swim test; OVX, ovariectomized; EE, enriched environment.

Increased locomotor activity is a robust phenotype in PACAP KO mice, but in the current study did not reveal an increased locomotor activity of KO mice in familiar HC. This, coupled with results from earlier work that showed elevated behavioral responses to novelty with increased exploration (Hashimoto et al., 2001), suggests that the hyperactive behavior was likely to be caused by an exploratory behavior toward novel stimuli. The hyperactive phenotype in a novel environment could be a confounding factor in other behavioral phenotypes, such as anxiety-like behavior, social behavior, and depression-like behavior.

Unlike the results in the current study, earlier work showed that PACAP KO mice displayed a normal startle response and impaired PPI, as well as increased immobility in the PS test (Tanaka et al., 2006; Hashimoto et al., 2009). These discrepancies might be due to differences in the experimental conditions and the background strains of the mice. In the previous research, PPI was carried out with a short acclimation period to the startle chamber (5 min) and different sound pressure levels of background white noise (65 dB) and prepulse stimuli (68, 71, 77 dB) in the startle response/PPI test. Under these conditions, PACAP KO mice on CD1 background strain showed a high degree (~70%) of PPI and significantly impaired PPI in the trials with large prepulse stimuli (Tanaka et al., 2006). On the other hand, the present study employed mice of a hybrid genetic background that included the C57BL/6J strain, which exhibits a naturally low level of PPI. Thus, the impairment of PPI reported earlier could not be observed in the current study. However, if large prepulse had been used in the F1 hybrid mice between the C57BL/6J and 129SvEv strains, the abnormality of PPI might have been detected. The PS test was performed in a large swimming pool (18.5 cm in diameter) with a deep water level (13 cm) in the work of Hashimoto and colleagues. These conditions could be more aversive than those of the present study. It is possible that the increased depression-like behavior could have been observed in the PACAP KO mice with hybrid genetic background, if a similar sized pool and water depth had been employed. In addition, the inconsistency in behavioral abnormalities observed between studies may be due to the gene × experience (i.e., breeding environment and test order) interaction. It would be interesting to assess the influence of gene × experience interactions on the test results by comparing naïve mice with mice subjected to the behavioral test battery in a future study. Such a study could provide new insights into genetic and/or environmental effects on the pathology of mental disorders.

Previous reports suggest that even small genetic variances may have a profound influence on behavioral phenotypes (Crawley et al., 1997; Matsuo et al., 2010). The difference in genetic background must therefore be considered in the interpretation of discrepancies in behavioral phenotype. For example, a mixed C57BL/6J and 129/Ola genetic background (Hashimoto et al., 2001) and a CD1 background (Tanaka et al., 2006; Hashimoto et al., 2007, 2009; Ishihama et al., 2010) were used in the earlier behavioral studies discussed herein. On the other hand, in the present study, a battery of behavioral tests was performed using C57BL/6J × 129SvEv F1 hybrid mice. Hybrid crosses can reduce the effects of flanking genes, which might be responsible for behavioral abnormalities ascribed to the null mutation (Silva et al., 1997; Crusio, 2004). In addition, F1 hybrid mice sometimes show beneficial phenotypes (e.g., longer life-span and better performance in terms of learning and memory) (Crawley et al., 1997). Indeed, PACAP KO mice have a normal life-span on a B6/129 F1 hybrid background, but show a high postnatal mortality rate on a single genetic background (Gray et al., 2001; Shintani et al., 2003). Moreover, PACAP plays important roles in neurogenesis during embryonic development (Falluel-Morel et al., 2007), and so, it is conceivable that the discrepancy in behavioral phenotypes may result from diversity of developmental processes induced by difference in background strains.

What are the mechanisms responsible for the behavioral abnormalities in PACAP KO mice? Consistent with PACAP KO mice, increased locomotor activity and abnormal anxiety-like behavior were also observed in PAC1 KO (Table 2), indicating that PACAP-PAC1-mediated signaling is involved in those behavioral abnormalities. However, forebrain neuron-specific PAC1 KO failed to exhibit these abnormal behaviors, suggesting that the abnormalities are forebrain neuron-independent. In addition, PACAP is expressed in broad regions of the brain, including the olfactory bulb, cerebral cortex, amygdaloid cortex, hippocampal formation, thalamus, and hypothalamus (Vaudry et al., 2000), and its expression is observed from an early developmental stage to adulthood (Vaudry et al., 2000). It is still unclear which brain regions and developmental stages are important for the behavioral phenotypes of PACAP KO mice, as conventional KO mice were used in previous studies as well as in our study. Future studies will be required to further determine the site(s) of the brain, developmental stage(s), and possible mechanism(s) that play important roles in the behavioral phenotypes observed in PACAP KO mice.

Human genetics studies have shown the association of the PACAP gene with psychiatric disorders, such as bipolar disorder (McInnes et al., 2001), schizophrenia (Hashimoto et al., 2007), depressive disorder (Hashimoto et al., 2010), and PTSD (Ressler et al., 2011). Intriguingly, duplications of the gene for VIPR2/VPAC2, which similarly binds to PACAP and a related peptide VIP, have been linked to significant risk for schizopherenia (Vacic et al., 2011). PACAP KO mice show remarkable behavioral abnormalities, supporting the idea that PACAP plays an important role in psychiatric disorders. For instance, increased locomotor activity displayed by PACAP KO mice is considered to be schizophrenia-like behavior in rodents, because psychostimulants induce schizophrenic-like symptoms in healthy individuals and increase locomotor activity in rodents (Tsai and Coyle, 2002; Chen et al., 2006; Powell and Miyakawa, 2006). Genetic association studies also demonstrate that genetic variants of the genes encoding PACAP or PAC_1_ are associated with schizophrenia, and the risk single nucleotide polymorphism (SNP) for the PACAP gene could be relevant to reduced hippocampal volume and/or poor memory performance, which are both neurobiological traits related to the risk for development of schizophrenia (Hashimoto et al., 2007). PACAP KO mice also showed mild performance deficit in working memory in this study. Anxiety-like behavior is associated with the sensitivity of the stress response, which is involved in the pathogenesis of schizophrenia and major depression (Turnbull and Bebbington, 2001; Goldberg and Fawcett, 2012). It is still inconclusive as to whether anxiety-like behavior is increased or decreased in PACAP KO mice. Given that increased exploration of the open area in the EP could potentially reflect an increased panic-like escape response to stress (Holmes et al., 2000; Miyakawa et al., 2003), we favor the idea that it is increased (see “Abnormal anxiety-like and slightly decreased depression-like behaviors in PACAP KO mice” in Results). If this is the case, it is consistent with the finding that SNPs spanning the PACAP gene are associated with PTSD (Ressler et al., 2011).

PACAP KO mice show increased social contact in sociability and social novelty preference test. PACAP KO mice also exhibited an increased number of contacts in a novel environment, although there was no significant difference in SI in the familiar HC between genotypes. Thus, there is a possibility that not only hyperactivity, but also increased social behavior is caused by enhanced novelty seeking, which is a trait related to ADHD (Anckarsäter et al., 2006). Patients with ADHD also have impaired working memory (Castellanos and Tannock, 2002), which is in line with the abnormal phenotype of PACAP KO mice in the RM and TM. These findings suggest that PACAP KO mice can serve as animal models of various mental disorders in addition to schizophrenia, such as major depressive disorder, generalized anxiety disorder, ADHD, and PTSD. Hence, these mice may prove useful for predicting unknown aspects of mental disorders and developing new therapeutic treatments.

### Conflict of interest statement

The authors declare that the research was conducted in the absence of any commercial or financial relationships that could be construed as a potential conflict of interest.
